# A simple, sensitive and non-destructive technique for characterizing bovine dental enamel erosion: attenuated total reflection Fourier transform infrared spectroscopy

**DOI:** 10.1038/ijos.2015.58

**Published:** 2016-03-25

**Authors:** In-Hye Kim, Jun Sik Son, Bong Ki Min, Young Kyoung Kim, Kyo-Han Kim, Tae-Yub Kwon

**Affiliations:** 1 Department of Dental Science, Graduate School, Kyungpook National University, Daegu, Korea; 2 Korea Textile Development Institute, Daegu, Korea; 3 Center for Research Facilities, Yeungnam University, Gyeongsan, Korea; 4 Department of Conservative Dentistry, School of Dentistry, Kyungpook National University, Daegu, Korea; 5 Department of Dental Biomaterials, School of Dentistry, Kyungpook National University, Daegu, Korea

**Keywords:** acidic beverage, enamel erosion, Fourier transform infrared spectroscopy, microhardness, sensitivity

## Abstract

Although many techniques are available to assess enamel erosion *in vitro*, a simple, non-destructive method with sufficient sensitivity for quantifying dental erosion is required. This study characterized the bovine dental enamel erosion induced by various acidic beverages *in vitro* using attenuated total reflection Fourier transform infrared (ATR-FTIR) spectroscopy. Deionized water (control) and 10 acidic beverages were selected to study erosion, and the pH and neutralizable acidity were measured. Bovine anterior teeth (110) were polished with up to 1 200-grit silicon carbide paper to produce flat enamel surfaces, which were then immersed in 20 mL of the beverages for 30 min at 37 °C. The degree of erosion was evaluated using ATR-FTIR spectroscopy and Vickers' microhardness measurements. The spectra obtained were interpreted in two ways that focused on the *ν*_1_, *ν*_3_ phosphate contour: the ratio of the height amplitude of *ν*_3_ PO_4_ to that of *ν*_1_ PO_4_ (Method 1) and the shift of the *ν*_3_ PO_4_ peak to a higher wavenumber (Method 2). The percentage changes in microhardness after the erosion treatments were primarily affected by the pH of the immersion media. Regression analyses revealed highly significant correlations between the surface hardness change and the degree of erosion, as detected by ATR-FTIR spectroscopy (*P*<0.001). Method 1 was the most sensitive to these changes, followed by surface hardness change measurements and Method 2. This study suggests that ATR-FTIR spectroscopy is potentially advantageous over the microhardness test as a simple, non-destructive, sensitive technique for the quantification of enamel erosion.

## Introduction

Enamel erosion caused by acids originating from acidic foods or beverages has attracted increasing attention in recent years because of the increased consumption of such drinks and foods.^1^ Several studies have reported a relationship between enamel erosion and the consumption of acidic beverages, such as carbonated drinks, fruit juices and sports drinks.^2, 3^ Many techniques have been used to investigate the loss of tooth substance during erosion, including micro- and nano-hardness techniques, profilometry, microradiography, chemical analysis, various microscopy techniques, secondary ion mass spectroscopy and quantitative light-induced fluorescence.^4^

Dental enamel is a highly mineralized tissue that is mainly composed of a ‘non-stoichiometric' form of hydroxyapatite (HAp). During enamel erosion by acidic beverages, acidic active agents in beverages interact with mineral crystals of the enamel.^5^ Softening of enamel surfaces is an early manifestation of erosion.^6^ Thus, the erosive potential of acidic agents on enamel surfaces can be simply quantified by microhardness measurements, which have been used extensively to investigate enamel erosion.^4^ Alteration of the surface chemical composition of erosive enamel is usually accompanied by changes in morphology and mechanical properties. However, such microhardness techniques provide no information on structural changes of enamel surface that occur at the molecular or atomic level during erosion.^7^ Although the microhardness technique has been used to compare the erosion of enamel by various solutions, the results reflect only the mechanical properties of the material.

Attenuated total reflection Fourier transform infrared (ATR-FTIR) spectroscopy has been demonstrated to be a very sensitive tool for the non-invasive study of molecular level changes in surface composition.^8^ Moreover, this technique permits repeated analyses of the same location on a surface, thus ensuring high comparability between spectra before and after sample treatment.^9^ Quantitative analysis using infared (IR) spectroscopy is based on the intensity of IR absorption being proportional to the magnitude of the change in the dipole moment of a bond during vibration. The band intensity of a particular functional group also depends on the number of groups that are present in the studied sample. Peak intensity (height) and area are most commonly used measurements in IR quantitative analysis. This technique has been used in tooth substrate research mainly to investigate the effects of sodium hypochlorite (NaOCl) or acid (for example, ethylenediamine tetracetic acid (EDTA)) on the surface chemistry of dentin.^10, 11, 12^ Amide bands I, II and III of collagen, and phosphate and carbonate bands of apatite have been identified in ATR-FTIR spectra of dentin. In studies of NaOCl-treated dentin, ATR-FTIR spectra are typically normalized to *ν*_3_ phosphate (*ν*_3_ PO_4_) peak intensity, and collagen depletion from dentin is then determined by the ratio of the absorbance peaks of amide I and *ν*_3_ PO_4_. When dentin is treated with acids such as EDTA, however, the *ν*_3_ PO_4_ peak intensity is insufficiently constant for normalization. Thus, ATR-FTIR was used to study the *ν*_3_ PO_4_ peak/amide I peak absorbance height ratio to investigate the decalcification of dentin surfaces after acid treatment.

To quantify enamel erosion using ATR-FTIR spectroscopy, a peak with constant intensity is required as an internal reference for spectral normalization. Enamel comprises a small proportion of organic matrix (<2%) and a large proportion of inorganic material (96%–98%). Although absorption bands attributed to amides I, II and III are observed in the ATR-FTIR spectra of enamel powder samples, these bands often overlap with other peaks (mainly CO_3_ peaks).^3^ Probably because of these difficulties, ATR-FTIR spectroscopy has not been extensively used to characterize enamel erosion.

The purpose of this *in vitro* study was to evaluate the effect of acidic beverages on the chemical structure of bovine tooth enamel by using ATR-FTIR spectroscopy. The IR spectra obtained were analysed in two ways, and both results were compared with microhardness data using regression analysis. In addition, field emission-scanning electron microscopy (FE-SEM) and X-ray diffraction (XRD) analysis were used to examine some of the test groups.

## Materials and methods

### Acidic beverages and pH and neutralizable acidity measurement

The acidic beverages used and their types, codes and manufacturers are listed in Table 1. Deionized water was used as a control. The pH of each beverage was measured using an electronic pH metre (SevenEasy pH; Mettler–Toledo GmbH, Schwerzenbach, Switzerland) at 37 °C on a heated magnetic stirrer. The pH metre was calibrated using test solutions of known pH prior to testing each of the beverages. Each beverage was tested using five different samples. The neutralizable acidity of each beverage was tested by placing 20 mL of liquid in a glass beaker, which was then placed in a water bath at 37 °C.^13^ Sodium hydroxide (0.1 mol̇L^−1^) was then gradually added to the sample, and the resulting increase in pH was continuously monitored until the sample reached neutrality. Each beverage was stirred continuously as the sodium hydroxide solution was added. The volume of sodium hydroxide required to increase the pH of the sample to neutrality was noted; this test was repeated five times for each beverage.

### Specimen preparation

For FTIR and microhardness analyses, 110 freshly extracted bovine anterior teeth were selected based on their dimensions, similarity in morphology and absence of any cracks or carious defects.^11^ After the roots were cut off, all teeth were stored in phosphate-buffered saline at 4 °C until use.^13^ Tooth plates (~8 mm × 8 mm × 3 mm) were prepared using a low-speed diamond saw under continuous water spray and were embedded in an acrylic resin. Each embedded tooth was wet-abraded to expose a flat enamel surface, which was then polished with silicon carbide paper (up to 1 200 grit) under water irrigation^14^ and then ultrasonicated in deionized water for 5 min to remove residual particles.^15^ The enamel samples were randomly allocated to 11 groups (10 specimens in each group) (Table 1) and were immersed in one of the solutions (20 mL) for 30 min^16^ under constant agitation (shaking table, 100 r·min^−1^) at 37 °C,^17^ rinsed with deionized water and finally blot-dried.

### ATR-FTIR spectroscopy

The alteration of the chemical structure of the enamel surfaces by the acidic beverages was investigated using a FTIR spectrophotometer (IRPrestige-21; Shimadzu, Kyoto, Japan) equipped with an ATR unit (MIRacle; Pike Technologies, Madison, WI, USA). Three spots were randomly chosen on the surface of each specimen, and the reverse side of the embedding resin was marked with a high-speed handpiece equipped with a fine diamond bur.^18^ The samples were placed onto the face of the germanium (Ge) crystal of the ATR unit with the marked surface facing up, and the tip of the micrometre clamp was pressed onto the marker to make the contact necessary to obtain a characteristic spectrum.^9^ This procedure ensured that the samples were measured at the same location before and after immersion in the beverages (*n*=5 per group).^8^ Absorbance spectra were acquired by scanning the specimens 100 times over a 750–4 000 cm^−1^ range at a resolution of 4 cm^−1^.^11^

**Figure 1:**
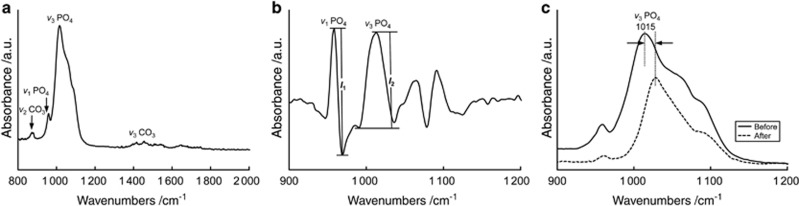
**Methods for quantifying the degree of erosion using ATR-FTIR spectroscopy**. (**a**) Baseline-corrected IR spectra in the region of 800–2 000 cm^−1^. (**b**) The peak-to-peak measurement technique applied to spectra that were initially second-derivative transformed and then multiplied by −1. The ratio of the height of *ν*_3_ PO_4_ (*l*_2_) to that of *ν*_1_ PO_4_ (*l*_1_) was calculated (Method 1). (**c**) A shift of the *ν*_3_ PO_4_ peak (~1 015 cm^−1^) to a higher wavenumber after erosion treatment was investigated (Method 2). ATR-FITR, attenuated total reflection Fourier transform infrared; a.u., arbitrary unit.

After baseline correction, the spectra were analysed in two ways (Figure 1). In Method 1, second-derivative spectra were calculated using add-on software (IRsolution 1.21; Shimadzu, Kyoto, Japan) and then multiplied by −1, focusing on the *ν*_1_, *ν*_3_ PO_4_ contour (900–1 200 cm^−1^).^19, 20^ The derivative values were determined using the peak-to-peak measurement technique; the ratio of the height amplitudes of *ν*_3_ PO_4_ (*l*_2_) to that of *ν*_1_ PO_4_ (*l*_1_) was calculated.^21^ In Method 2, the shift of the *ν*_3_ PO_4_ peak at ~1 015 cm^−1^ to higher wavenumbers was investigated.^7^

### Microhardness measurements

The Vickers' microhardness (VMH) of the enamel surfaces was measured using a microhardness tester (HMV-2; Shimadzu, Kyoto, Japan) before and after immersion in each beverage. Indentations obtained using a 200-g force for 10 s were obtained at five different sites on each sample and observed under a magnification of × 400;^22^ the measurements collected after the erosion treatment were recorded near the baseline indentations (100 μm space).^23^ The VMH of each specimen was recorded as the average of the five readings, and the results were transformed to a percentage value (in which the baseline value was 100%, and the new value was calculated as a percentage of the baseline (*n*=5 per group)).^18^

### Field emission-scanning electron microscopy

For three selected test groups (DW, CC and YA), one representative sample was prepared as described above for the FE-SEM (JSM-6700F; Jeol, Tokyo, Japan) analysis. The enamel samples were dried using a series of ethanol solutions and then sputter-coated with platinum. Photographs of representative areas of the surfaces were taken at × 4 000 magnification.

### XRD analysis

For the DW, CC and YA groups, XRD (XRD-7000; Shimadzu, Kyoto, Japan) was used to characterize the crystalline phase of one representative sample of each group; the XRD used Cu *K*_α_ radiation (*λ*≈0.1540 6 nm) at an operating condition of 40 kV and 30 mA with a scan range of 10°<2*θ*<70°. The phases of the samples were identified using spectra of known phases from the International Centre for Diffraction Data (ICDD) database. To compare the difference in peak intensity for the (0 0 2) face between the samples, the intensity ratio of the diffraction peaks corresponding to the (0 0 2) and (1 1 2) faces was calculated (*n*=3 per group).^24^

### Statistical analysis

Each data set was normally distributed (Shapiro–Wilk test) and exhibited equal variances (Levene test); therefore, a one-way analysis of variance (ANOVA) and Duncan's *post hoc* test were used to evaluate the data.^25^ Fifty-five comparisons were possible among the 11 experimental groups. For each interpretation or measurement (Methods 1 and 2 and surface hardness change), the ratio of comparisons indicating significant differences to the total number of possible comparisons was reported as the testing sensitivity.^26^ When appropriate, linear or logarithmic regression analyses were performed to correlate the degree of erosion calculated using Methods 1 or 2 with the surface hardness change. Statistical analyses were performed using SPSS 17.0 for Windows (SPSS, Chicago, IL, USA) at a significance level of 0.05.

## Results

The pH and neutralizable acidity of the studied beverages are summarized in Table 1. Overall, the carbonated beverages had lower pH values, whereas the yogurt drinks had higher pH values. The energy drinks and yogurt drinks generally exhibited high-neutralizable acidity. Particularly in the case of carbonated beverages and fruit juices, neutralizable acidity varied greatly even within a beverage type.

**Figure 2:**
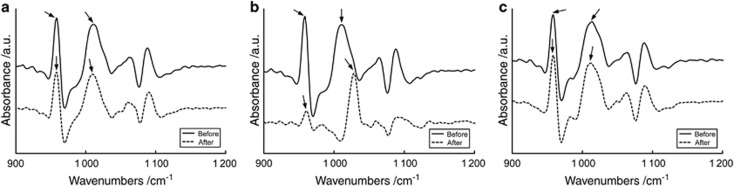
**Representative spectra that were initially second-derivative transformed and then multiplied by −1 before (solid line) and after (dotted line) erosion treatment.** (**a**) DW; (**b**) CC; (**c**) YA. The arrows indicate *ν*_1_ (left) and *ν*_3_ (right) PO_4_ regions. a.u., arbitrary unit; CC, coca-cola classic; DW, deionized water; YA, activia drinks plain.

Figure 2 shows the representative ATR-FTIR spectra of enamel after erosion treatment obtained through Method 1. No distinct amide bands were observed in the spectra. Compared with the DW group, the CC group showed a greater change in the *l*_2_ (*ν*_3_ PO_4_)/*l*_1_ (*ν*_1_ PO_4_) ratio than did the YA group.

**Figure 3:**
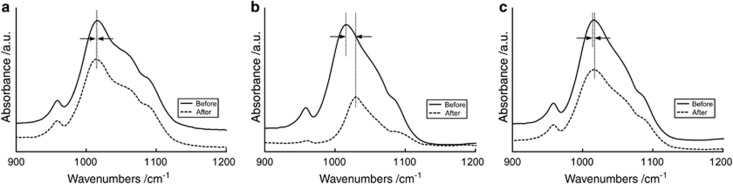
**Representative spectra showing the shift of the *ν*_3_ PO_4_ peak to higher wavenumbers after erosion treatment.** (**a**) DW; (**b**) CC; (**c**) YA. a.u., arbitrary unit; CC, coca-cola classic; DW, deionized water; YA, activia drinks plain.

The changes in the position of the *ν*_3_ PO_4_ peak before and after erosion treatment (Method 2) are shown in Figure 3. In the DW group, the peak was shifted only slightly. In CC, in contrast, the peak shifted distinctly to a higher wavenumber (~10 cm^−1^). Only a slight shift of the peak was detected in the YA group.

**Figure 4:**
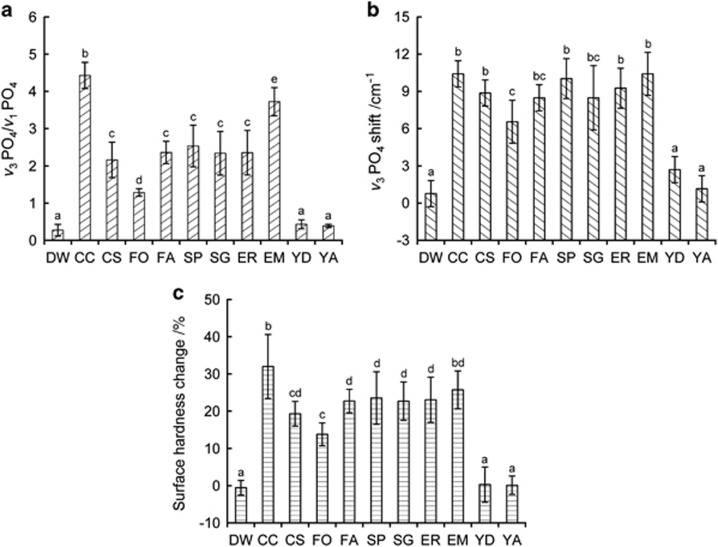
**Degree of erosion and percentage microhardness change.** (**a**, **b**) Degree of erosion calculated *via* Method 1 (**a**, *ν*_3_ PO_4_/*ν*_1_ PO_4_) and *via* Method 2 (**b**, *ν*_3_ PO_4_ peak shift) based on ATR-FTIR spectroscopy. (**c**) Percentage microhardness change after erosion treatment. Vertical bar=±1 standard deviation. In each figure, similar lower-case letters indicate statistically equivalent values (*P*>0.05). ATR-FITR, attenuated total reflection Fourier transform infrared; CC, coca-cola classic; CS, sprite; DW, deionized water; EM, monster energy; ER, red bull; FA, minute maid punch apple holic; FO, minute maid premium orange 100; SG, gatorade lemon; SP, poweraid mountain blast; YA, activia drinks plain; YD, Denmark drinking yogurt plain.

Figure 4 depicts the degree of erosion calculated through Methods 1 and 2. The percentage microhardness changes for each group are also shown. Overall, both methods based on ATR-FTIR spectroscopy exhibited similar trends, and the CC and YA groups showed the highest and lowest degrees of erosion, respectively. Except for the yogurt drinks, all acidic beverages induced significant microhardness changes after erosion treatment (*P*<0.05). The degrees of erosion that were measured through Methods 1 and 2 showed sensitivities of 0.76 (42/55) and 0.44 (24/55), respectively. However, the sensitivity for surface hardness change was 0.56 (31/55).

**Figure 5:**
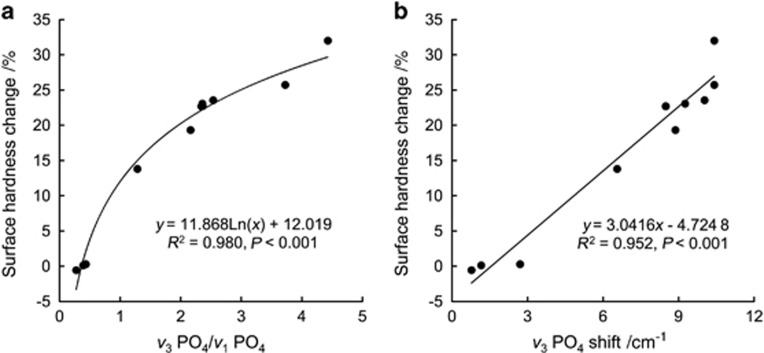
**Graphs of the logarithmic and linear regression of the percentage surface microhardness change as a function of the degree of erosion.** (**a**) Calculated via Method 1 (*ν*_3_ PO_4_/*ν*_1_ PO_4_); (**b**) via Method 2 (*ν*_3_ PO_4_ peak shift) based on ATR-FTIR spectroscopy. ATR-FITR, attenuated total reflection Fourier transform infrared.

Graphs of the regression of percentage of surface microhardness change as a function of the degree of erosion calculated via Methods 1 and 2, which are based on ATR-FTIR spectroscopy, are presented in Figure 5. The surface hardness change increased logarithmically and linearly with the degree of erosion as measured through Methods 1 and 2, respectively.

**Figure 6:**
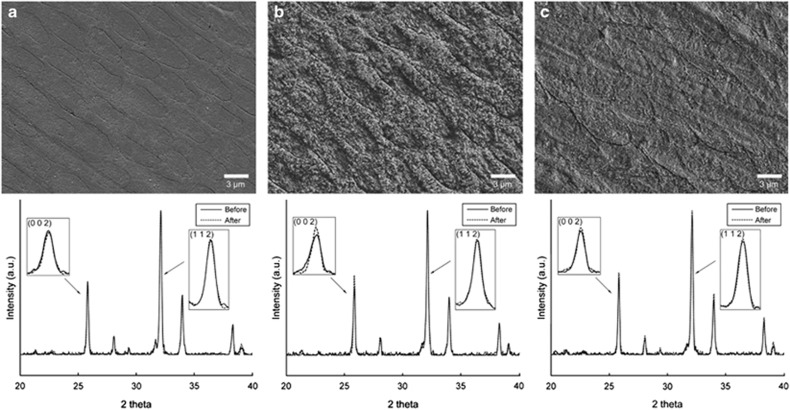
**Representative FE-SEM images of eroded enamel surfaces and XRD spectra of enamel surfaces before and after erosion treatment for three selected test groups.** (**a**) DW; (**b**) CC; (**c**) YA. All diffraction peaks were assigned to hydroxyapatite (the standard ICDD card no. 09-432). Upper, × 4 000; bar, 3 μm. a.u., arbitrary unit; CC, coca-cola classic; DW, deionized water; FE-SEM, field emission-scanning electron microscopy; ICDD, International Centre for Diffraction Data; XRD, X-ray diffraction; YA, activia drinks plain.

Figure 6 shows representative FE-SEM images and XRD patterns of eroded enamel surfaces for three selected test groups. The images clearly show the loss of tooth substance induced by acidic beverages, in particular by CC (Figure 6b). All peaks closely resembled the diffraction peaks of HAp in the ICDD database (ICDD no. 09-432). The diffraction peak intensity ratio of the (0 0 2) face to the (1 1 2) face was significantly higher in the CC group than in the YA group (1.23±0.03 *vs* 1.01±0.01, respectively; ANOVA and Duncan's *post hoc* test, *P*<0.05).

## Discussion

ATR-FTIR spectroscopy is a highly sensitive tool for the non-invasive study of changes in surface composition at the molecular level and enables easy characterization with little or no sample preparation. In this study, the degree of erosion of bovine tooth enamel induced by acidic beverages was quantified using ATR-FTIR spectroscopy. The resulting spectra were analysed in two ways, focusing on the *ν*_1_, *ν*_3_ PO_4_ contour. Both results (Methods 1 and 2) showed highly significant correlations with the percentage microhardness change according to regression analyses (Figure 5).

The mineral in dental enamel is a calcium-deficient carbonated HAp containing fluoride. A simplified formula for tooth mineral composition is Ca_10−*x*_Na_*x*_(PO_4_)_6−*y*_(CO_3_)_*z*_(OH)_2−*u*_ F_*u*_;^27^ thus, enamel differs from ‘stoichiometric' HAp, which has the formula (Ca_10_(PO_4_)_6_(OH)_2_).^5^ The ATR-FTIR spectra of the enamel surface was consistent with the results obtained in previous studies (Figure 1a).^3, 28^ A very strong peak was observed at ~1 015 cm^−1^, which was attributed to the phosphate (PO_4_) anti-symmetric stretching mode (*ν*_3_). The phosphate symmetric stretching mode (*ν*_1_ PO_4_) appeared at ~962 cm^−1^. Strong peaks assigned to B-type carbonate substitution (carbonate substitution for the phosphate ion) were observed at 872 cm^−1^ (*ν*_2_ CO_3_) and at 1 405 and 1 450 cm^−1^, respectively (*ν*_3_ CO_3_).^29^ Substitutions (especially by carbonate) in the mineral crystal lattice weaken enamel structure, rendering the mineral more acid-soluble than HAp.^30^

In this study, the percentage change in microhardness after erosion treatment was primarily affected by the pH values of the immersion media (Table 1, Figure 4c). Enamel erosion begins with surface softening. This process is followed by a continuous, layer-by-layer dissolution of the enamel crystals, leading to permanent loss of tooth volume with a softened layer at the surface of the remaining tissue. However, the microhardness results showed that pH is not the only factor determining the degree of erosion, although CC (the lowest pH, Table 1) and YA (the highest pH) produced the greatest and smallest surface microhardness changes, respectively. The erosive potential of an acidic beverage does not depend exclusively on pH but is also strongly influenced by the acidic content of the product, which is best measured as neutralizable acidity.^31^ The wide variation in neutralizable acidity is probably due to the differing levels of citric, lactic and malic acids in the various beverages.^32^ In this study, all acidic beverages tested, except for EM, exhibited low neutralizable acidity values (<20 mL of 0.1 mol·L^−1^ sodium hydroxide, Table 1). Previous studies have shown that in most instances, acidic pH combined with high neutralizable acidity results in high levels of erosion.^32^ This was certainly the case for EM, which caused erosion.

The results of previous studies have suggested that an IR peak near 1 015 cm^−1^ is indicative of acid-induced alteration of the atomic bonding in superficial enamel apatite.^7, 11, 33^ In this study, the intensity of the *ν*_1_ PO_4_ peak markedly decreased after the erosion treatment. The intensity of the *ν*_3_ PO_4_ peak was relatively unaffected; however, the peak was shifted to higher wavenumbers after the erosion treatment, indicating that the P–O bond length was reduced due to erosion. Thus, the ATR-FTIR spectra could be interpreted in two ways, focusing on alterations in either the *ν*_1_ or *ν*_3_ PO_4_ peak (Methods 1 and 2).

Method 1 (Figures 1b and 2) analysed the ratio of the height of *ν*_3_ PO_4_ (*l*_2_) to that of *ν*_1_ PO_4_ (*l*_1_), with the former and the latter being assigned to non-stoichiometric and stoichiometric forms of HAp, respectively. Because the intensity of the *ν*_1_ PO_4_ peak was not sufficient for quantification, the spectra were transformed on the basis of the second derivative. A second-derivative spectrum narrows sharp peaks and flattens broad peaks while preserving quantitative information. After inversion, the spectrum was multiplied by −1. In Raman spectra of tooth enamel, the strongest peak (960 cm^−1^) is attributed to *ν*_1_ PO_4_, and the bands at 1 045 and 1 024 cm^−1^ are assigned to *ν*_3_ PO_4_.^8^ The intensity of the *ν*_1_ PO_4_ band in Raman spectroscopy is linearly proportional to phosphate group concentration within the HAp molecule.^18^ As shown in Figure 2b, erosion treatment changed *l*_1_, which is related to *ν*_1_ PO_4_, more than *l*_2_, which is related to *ν*_3_ PO_4_. Thus, for Raman spectroscopy, the intensity of the *ν*_1_ PO_4_ peak in second-derivative ATR-FTIR spectra appears to provide important information for the quantification of enamel erosion *in vitro*.

Using Method 2 (Figures 1c and 3), we found that the erosion treatment induced a shift of the anti-symmetrical *ν*_3_ PO_4_ stretching mode to a higher wavenumber in the ATR-FTIR spectra, indicating stronger bonding of the corresponding O atoms to the P atoms.^33^ In apatite, each P atom is linked to four Ca atoms *via* a shared oxygen atom; that is, the framework comprises P–O–Ca atomic bridges.^33^ Hence, a shortening of P–O bonds necessitates lengthening of the adjacent Ca–O bonds due to the redistribution of the electron density of states in the vicinity of the bridging oxygen.^7^ Weakened Ca–O bonds increase the release of Ca from enamel upon erosion.^33^ Thus, exposure to acidic beverages lengthens or breaks the Ca–O bonds and consequently strengthens the P–O bond, as seen in the shift of the *ν*_3_ PO_4_ peak in the IR spectra.

The sensitivity of each technique (Methods 1 and 2 and the microhardness test) was measured to determine the extent to which each procedure could to detect differences among the 11 groups tested. The sensitivity to differences was greatest for Method 1, followed by the measurement of surface hardness change and Method 2 (Figure 4), indicating that Method 1, which is based on ATR-FTIR spectroscopy, is potentially advantageous over microhardness testing for the quantification of enamel erosion. Moreover, logarithmic regression analysis (Figure 5a) showed that the slope of the prediction line of the surface hardness change increased only logarithmically with increasing erosion for Method 1. Thus, Method 1 was much more sensitive than microhardness measurement for greater enamel erosion. In contrast, Method 2, which is based on the IR peak shift, was less sensitive than microhardness measurement, probably due to the limited resolution of the IR spectra.

In this study, groups CC and YA consistently showed the highest and lowest degree of erosion among all analytical methods (Figure 4). The DW group produced virtually no erosion on the enamel surface. The obtained FE-SEM surface images (Figures 6a–6c, upper) indicated that erosion occurred predominantly along the enamel prism peripheries rather than in the prism cores (in particular in the CC group). This finding was supported by the results of the XRD analysis (Figures 6a–6c, lower). The peak intensity ratio of the (0 0 2) face to that of the (1 1 2) face was stronger in the CC group than in the DW and YA groups, indicating that HAp crystals in ‘prism' peripheries were removed preferentially over those in the ‘prism' cores during erosion, such that the degree of orientation of the HAp crystals was enhanced along the *c* axis.^34^

The present *in vitro* study supports the use of ATR-FTIR spectroscopy for the simple, non-destructive evaluation of dental enamel erosion. The degree of erosion on enamel surfaces induced by various acidic beverages was easily quantified through the *ν*_3_ PO_4_/*ν*_1_ PO_4_ height amplitude ratio or the *ν*_3_ PO_4_ peak shift in ATR-FTIR spectra. Erosion treatment thus appeared to shorten the P–O bond in *ν*_3_ PO_4_ (at ~1 015 cm^−1^) when calcium ions were driven from the apatite structure without a significant change in the bond number. However, a significant reduction in the intensity of the *ν*_1_ PO_4_ signal (at ~962 cm^−1^) implied an alteration in the structure of stoichiometric apatite during erosion by acidic beverages. Based on a sensitivity analysis, ATR-FTIR spectroscopy (in particular, when analysed using Method 1) appears to be advantageous over microhardness testing as a simple, sensitive and non-destructive technique for the quantification of enamel erosion.

However, caution should be used when applying the ATR-FTIR techniques used in this study directly to clinical applications, because of certain limitations related to the experimental design. First, ATR-FTIR spectroscopy appears more suitable for characterizing superficial enamel erosion rather than deep enamel defects, due to its limited penetration depth (1–10 μm).^35^ Second, bovine teeth were used as a representative substitute of human teeth (the chemical structure and response to erosive challenges of bovine enamel are similar to those of human enamel).^36^ Third, the bovine enamel was ground flat to simplify measurement procedures. Because the inner enamel is known to be more soluble than surface enamel, erosion occurs more rapidly in polished samples.^4^ Finally, the influence of saliva and plaque/pellicle on enamel erosion was not addressed in this study. Enamel that is softened by acidic beverages can re-harden following exposure to saliva. In addition, dental plaque/pellicle may provide a significant level of protection to tooth enamel against dental erosion by acidic beverages.^37^ Thus, the degree of erosion (Figure 4) might be lower in the intraoral environment.

## Conclusion

The degree of enamel surface erosion induced by various acidic beverages was easily quantified through the *ν*_3_ PO_4_/*ν*_1_ PO_4_ height amplitude ratio (Method 1) or the *ν*_3_ PO_4_ peak shift (Method 2) in ATR-FTIR spectra. Therefore, erosion treatment appears to shorten the P–O bond in *ν*_3_ PO_4_ (at ~1 015 cm^−1^) when calcium ions are driven off the apatite structure without significant change in the bond number. However, a significant reduction in the intensity of the *ν*_1_ PO_4_ signal (at ~962 cm^−1^) implies an alteration in the structure of stoichiometric apatite during erosion by acidic beverages. Based on a sensitivity analysis, ATR-FTIR spectroscopy (in particular, Method 1) appears to be advantageous over microhardness testing as a simple, sensitive and non-destructive technique for the quantification of enamel erosion.

## Figures and Tables

**Table 1:** Beverages used and their pH and neutralizable acidity

Type	Brand name (code)	Manufacturer	pH^*^	Neutralizable acidity^*^/mL
Deionized water	(DW)	—	6.79±0.10	0.004±0.001
Carbonated beverage	Coca-Cola Classic (CC)	Coca-Cola Enterprises, Yangsan, Korea	2.46±0.03	7.82±0.31
	Sprite (CS)	Coca-Cola Enterprises, Yangsan, Korea	3.04±0.03	13.26±0.76
Fruit juice	Minute Maid Premium Orange 100 (FO)	Coca-Cola Enterprises, Namwon, Korea	3.73±0.03	15.52±0.82
	Minute Maid Punch Apple Holic (FA)	Coca-Cola Enterprises, Namwon, Korea	2.95±0.04	6.03±0.41
Sports drink	Poweraid Mountain Blast (SP)	Coca-Cola Enterprises, Namwon, Korea	3.24±0.03	6.49±0.21
	Gatorade Lemon (SG)	Gatorade, Lotte Chilsung Beverage, Anseong, Korea	3.32±0.04	7.43±0.39
Energy drink	Red Bull (ER)	Red Bull, Vienna, Austria	3.45±0.04	16.48±0.70
	Monster Energy (EM)	Monster Beverage, Corona, CA, USA	3.49±0.06	21.47±0.73
Yogurt drink	Denmark Drinking Yogurt Plain (YD)	Dongwon Dairy Foods, Jeongeup, Korea	4.20±0.05	15.43±0.34
	Activia Drinks Plain (YA)	Danone Korea, Muju, Korea	4.29±0.04	15.61±0.56

Within each parameter, similar lower-case superscripted letters indicate statistically equivalent values (*P*>0.05).
